# Is knowledge about COVID-19 associated with willingness to receive vaccine, vaccine uptake, and vaccine booster uptake in rural Malang, Indonesia?

**DOI:** 10.3389/fpubh.2023.1203550

**Published:** 2023-06-07

**Authors:** Sujarwoto Sujarwoto, Asri Maharani

**Affiliations:** ^1^Department of Public Administration, Faculty of Administrative Science, Brawijaya University, Malang, Indonesia; ^2^Department of Nursing, Faculty of Health and Education, Manchester Metropolitan University, Manchester, United Kingdom

**Keywords:** COVID-19 knowledge, willingness to receive vaccine, vaccine uptake, vaccine booster uptake, vaccine hesitancy

## Abstract

**Background:**

Lack of knowledge regarding the coronavirus disease (COVID-19) and COVID-19 vaccines is a key barrier to COVID-19 vaccine uptake in low- and middle-income countries (LMICs).

**Aims:**

To examine factors associated with knowledge about COVID-19 and the association between knowledge of COVID-19, willingness to receive a COVID-19 vaccine, and vaccine uptake in Malang, East Java, Indonesia.

**Method:**

A cross-sectional study among individuals aged 15–99 years was conducted in Malang, Java Timur, Indonesia between November 2022 and January 2023. Of 10,050 potential respondents, 10,007 were able to complete the survey. The main independent variable was knowledge about COVID-19, which was assessed using a six-item questionnaire. The dependent variables were COVID-19 vaccine uptake and COVID-19 booster vaccine uptake. The mediating variable was respondent’s willingness to receive a COVID-19 vaccine. Linear regression was used to examine factors associated with knowledge about COVID-19. Logistic regression was employed to examine the association of knowledge about COVID-19 with vaccine uptake. Generalized structural equation modeling (GSEM) was performed to examine whether willingness to receive a vaccine mediated the association between knowledge about COVID-19 and vaccination uptake.

**Findings:**

The percentage of respondents who reported having received at least one dose of a COVID-19 vaccine was 94.8%, while the percentage of those who reported having received at least three doses was 88.5%. These numbers are higher than the national average for COVID-19 vaccine and booster vaccine uptake. Most respondents answered about four of six knowledge items correctly (*M* = 4.60, SD = 1.1). Among respondents who had not received a vaccine, 83.1% expressed willingness to receive a vaccine when it became available to them. Older, more educated, employed respondents, and those with higher economic status, demonstrated more accurate knowledge about COVID-19 than younger, less educated, unemployed respondents and those with lower economic status. Respondents who demonstrated more accurate knowledge about COVID-19 were more likely to have received a vaccine (OR = 1.528, 95% CI = 1.428–1.634) and a booster vaccine (OR = 1.260, 95% CI = 1.196–1.328). Willingness to receive a vaccine mediated the association between knowledge about COVID-19 and vaccine uptake (coef. = 0.426, 95% CI = 0.379–0.473).

**Implications:**

Interventions and public health programs aiming to improve knowledge about COVID-19 can be implemented to improve individual willingness to receive COVID-19 vaccination and to improve COVID-19 vaccine uptake among the general population.

## 1. Introduction

COVID-19 vaccine hesitancy is one of the major barriers to vaccine uptake worldwide (1). This barrier is often associated with a lack of knowledge regarding COVID-19 and COVID-19 vaccines within the general population. It is well documented that lack of knowledge regarding COVID-19 vaccines, vaccination schedules, location of vaccination centers, and vaccine effectiveness leads to lower vaccine uptake (2). One study also reported that more accurate knowledge about COVID-19 vaccines is associated with lower levels of hesitancy and higher levels of vaccination acceptance (1). In contrast, less accurate knowledge and misinformation regarding COVID-19 vaccines are the main drivers of vaccine hesitancy (3).

While studies examining the role of knowledge regarding COVID-19 vaccines in vaccine hesitancy have been widely conducted, the number of studies considering the link between knowledge about COVID-19 and vaccine uptake and booster vaccine uptake remains limited (4). The literature on health literacy highlights that health knowledge constitutes a background factor that promotes health prevention activities, including vaccination uptake (5). This concept is supported by a study that suggests that knowledge supports effective health-related decision-making (6). People with more knowledge about health risks, signs and symptoms, and the benefits of preventive actions tend to have healthier lifestyles (7). A higher level of health knowledge is also associated with less difficulty in navigating the health care system, greater access to health care, and more effective utilization of health resources for disease prevention (8). This concept is consistent with the expression “knowledge is power,” which has appeared in cognitive science for decades to illustrate the importance of knowledge in human and artificial intelligence (9). Theories such as the long-term working memory theory propose that the advantages conferred by knowledge are due to knowledge structures that facilitate comprehension of and memory for information that is germane to the knowledge domain (8).

Moreover, studies on communicable diseases have shown that knowledge about a disease is an important predictor of behaviors that impact the spread of the disease (8, 10). For example, prior knowledge about a disease has been shown to encourage protective behaviors such as increased handwashing and increased willingness to forgo public activities (10). Misunderstanding or knowledge deficit regarding influenza has also been shown to reduce the adoption of protective behaviors (11). However, a recent study on COVID-19 reported no effects of knowledge on protective behavior (12). Another study, conducted when physical distancing but not mask-wearing was highly recommended (13), found that higher levels of COVID-19 knowledge were associated with attending fewer large gatherings but not with wearing a mask when leaving home (14). These mixed findings indicate a need for further investigation in various health system contexts, especially in LMICs, where knowledge sources regarding diseases are limited, low uptake of vaccination often occurs, and most people are less educated and less willing to accept vaccines (15, 16).

Individuals with chronic disease, including cardiovascular disease and chronic obstructive pulmonary disease, are among the target of COVID-19 vaccination strategies because they are more likely to have the SARS-Cov-2 infection, and once infected, they are at higher risks of developing serious complications that can lead to mortality (17, 18). Mohseni et al. found that influenza vaccination among patients with heart failure was associated with a lower risk of hospitalization in England (19). However, the coverage of individuals with chronic disease is more likely to be lower. For example, a study in Italy showed that only 22.8 and 7.2% of patients with chronic diseases and hospitalized due to those conditions received influenza and pneumococcal vaccines, respectively (20, 21). A meta-analysis study including data from 31 studies from countries found that the pooled acceptance rate of COVID-19 vaccine among patients with chronic diseases is 65% (22), which is still below the target (75–90%). Improving the vaccine acceptance of patients with chronic diseases is thus important to reduce hospitalization and mortality.

The case of Indonesia is interesting to examine the role of general population knowledge about COVID-19 in vaccination uptake and booster vaccination uptake. The country has faced some difficult COVID-19 surges, with more than 6.41 million COVID-19 cases and 157,844 deaths as of September 2022, making vaccination uptake crucial to reduce morbidity and mortality. By March 18, 2023, the proportion of the Indonesian population having received at least one dose of COVID-19 vaccine rose to 86% and those with at least three doses comprised 37% of the population (23). Moreover, the percentage of vaccination uptake in Malang, the location of this study, is much higher than the national vaccination coverage with 2,534,372 (91.2%) having received a first dose and 2,416,046 (87.8%) a second dose (24).

Accordingly, this study has four aims: (1) to investigate knowledge regarding COVID-19, willingness to receive a COVID-19 vaccine, vaccine uptake, and booster vaccine uptake among the general population in Malang, Indonesia; (2) to examine sociodemographic determinants of knowledge regarding COVID-19 among the general population in the district; (3) to examine the association of knowledge about COVID-19 on vaccine uptake and booster vaccine uptake among the general population in the district; and (4) to examine whether willingness to receive a vaccine mediates the linkage between knowledge regarding COVID-19 and vaccination uptake among the general population in the district.

Our main hypotheses are that individuals with more accurate knowledge regarding COVID-19 can better understand the disease and its risks, signs, and symptoms and are therefore more willing to receive a vaccine and more likely to do so. Likewise, individuals with less accurate knowledge regarding COVID-19 are more likely to misunderstand the disease or lack knowledge about it, rendering them less willing to accept a vaccine and less likely to receive one. Although this study focuses on Malang District, Indonesia, we hope that our findings will not only aid in designing and developing educational interventions specifically targeted to improve COVID-19 vaccine uptake in the district but also beyond the study location, especially in LMICs with similar health contexts.

## 2. Methods

### 2.1. Study location

This study was conducted in the district of Malang, Jawa Timur, Indonesia, from November 01, 2022, to January 25, 2023, when the government of Indonesia declared COVID-19 an epidemic disease. The COVID-19 booster vaccination program had already been launched in the district. Malang is the second-largest district in East Java Province, with a 2022 population of 2,751,761 people distributed across 390 villages (273 or 70% of which are rural and 117 or 30% of which are urban). Malang has 39 primary healthcare centers (one for every 65,000 people) and 390 village health posts (one for every 7,000 people). Malang classifies 10.15% of its population as “poor or near poor,” compared to 11.46% in all of East Java province (25). The Malang authority carried out its first COVID-19 vaccination program in January 2021 with 2 million doses of vaccine. It is reported that 2,589,407 people have been vaccinated (94.1%) (24). The second and third phases of the vaccination campaign in Malang were carried out in May 2021 and January 2022, with 5.6 million doses of vaccines reaching 2,534,372 individuals (91.2%) and 2,416,046 individuals (87.8%), respectively (18). The percentage of vaccination uptake for the first and second doses in Malang is higher than the national vaccination uptake, which is reported at 86% for the first dose and 57% for the second dose (24).

### 2.2. Study design and sampling method

This cross-sectional study was conducted among individuals aged 15–99 years. The sampling population was determined using a stratified sampling design, with the population stratified into urban and rural areas. Based on a confidence level of 99.0% and a margin of error of 2%, we found the minimum samples for rural and urban areas to be 4,151 and 4,139 respondents, respectively. Initially, 10,050 potential respondents (5,049 in rural areas and 5,001 in urban areas) provided written informed consent and agreed to participate in this study. To encourage participants to participate in the survey, we provided a door prize for 100 randomly selected participants at the end of the survey. Of 10,050 potential respondents, 10,007 were able to complete the survey.

### 2.3. Data collection process

Before data collection, a pretest of the questionnaire was conducted in urban and rural villages at Mojokerto East Java from 12–20 August 2022. The pretest focused on questionnaire content, field editing protocols, the use of data collection apps, and general field procedures. The results of the pilot survey indicated that all respondents could easily understand the questions. Overall, the survey took 30 to 40 min to complete. We used KoboToolbox (a simple, robust, and powerful data collection app) to generate a questionnaire (26). The survey apps were used by 160 trained field researchers in charge of data collection. All recruited field researchers underwent a thorough 2-day training to learn and practice using the survey app.

**Figure 1:**
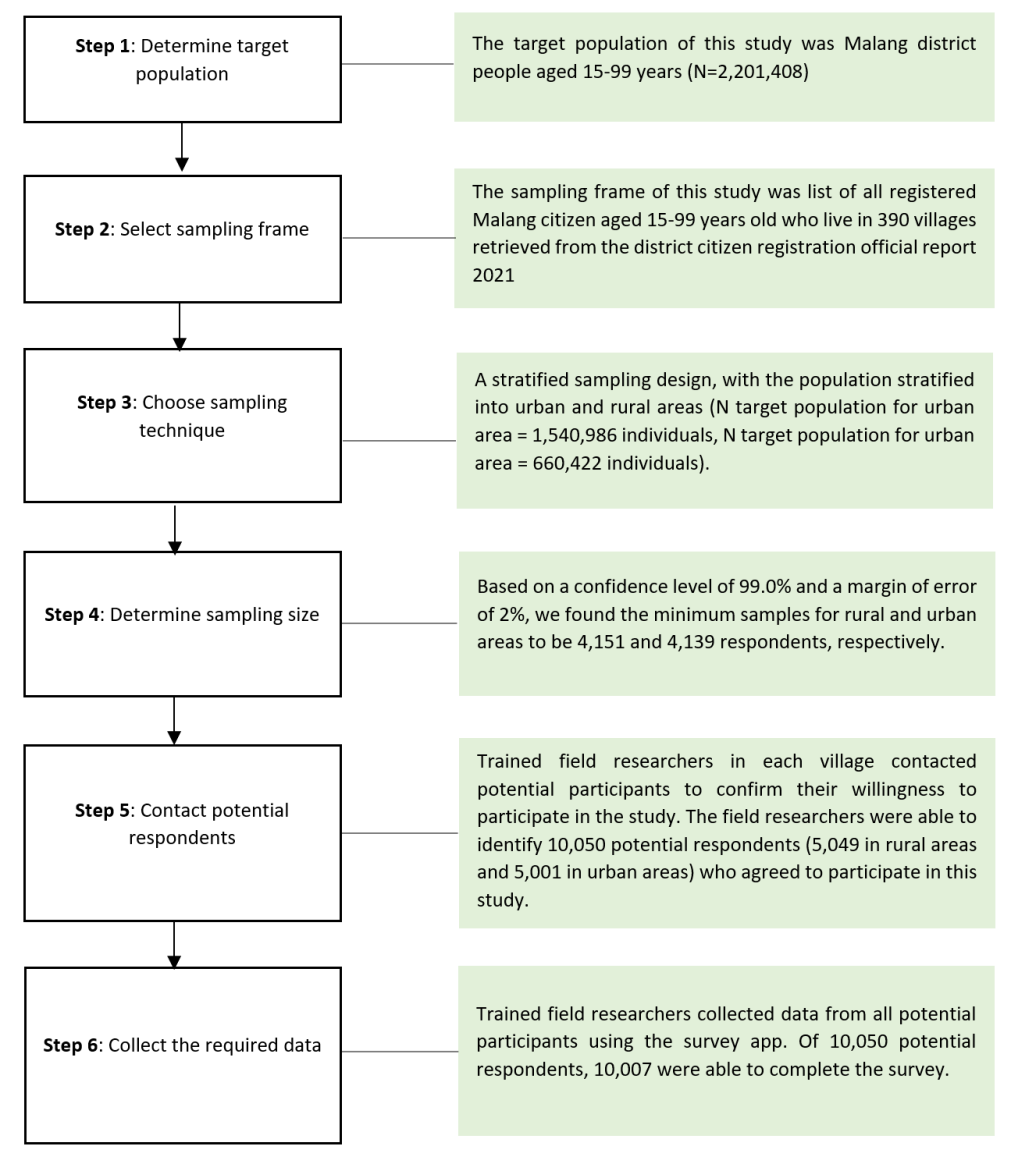
Workflow of data collection in this study.

Figure 1 describes the data collection process in this study. The target population of this study was Malang district people age 15–99 years (*N* = 2,201,408). The sampling frame of this study was a list of all registered Malang citizens aged 15–99 years who live in 390 villages retrieved from the district citizen registration official report 2021. We applied a stratified sampling design, with the population stratified into urban and rural areas (N target population for urban area = 1,540,986 individuals, N target population for urban area = 660,422 individuals). Based on a confidence level of 99.0% and a margin of error of 2%, we found the minimum samples for rural and urban areas to be 4,151 and 4,139 respondents, respectively.

Trained field researchers in each village contacted potential participants to confirm their willingness to participate in the study. The field researchers were able to identify 10,050 potential respondents (5,049 in rural areas and 5,001 in urban areas) who agreed to participate in this study. Sixth, collecting data: trained field researchers collected data from all potential participants using the survey app. Written informed consent was obtained from all respondents before data collection. Prior to interviewing, respondents were informed about the importance of participating in the survey. Confidentiality and anonymity were also ensured during data collection. Due COVID-19 pandemic situation, the field researchers were equipped with several items of personal protective equipment for COVID-19 protection, including medical masks (N95 3 M Type 9,010), face shields (headgear with clear visor), surgical gloves (Golden Glove latex), and hand sanitizer. Of 10,050 potential respondents, 10,007 were able to complete the survey.

Quality control was done in the field as well as in the University of Brawijaya office. In the field, it was the responsibility of the supervisor and data editor to listen to the recording interview for selected random interviews. In the first two enumeration areas, they had to listen to up to two interviews of each field researcher and thereafter randomly selected interviews. Supervisors also had the responsibility to do observation and verification of 10% of interviews. Verification was done by listening to parts of interview recordings. We also had a team of people in the University of Brawijaya office who listened to random parts of these recordings for random interviews and then compared answers to the electronic data. When discrepancies were found they got back to the teams, generally within the first week of the original interview for field researchers to re-check questionable answers.

### 2.4. Measures

The dependent variables in this study were COVID-19 vaccine uptake and COVID-19 booster vaccine uptake. COVID-19 vaccine uptake was measured using the question: “Have you received at least one dose of a COVID-19 vaccine as of today?.” Likewise, COVID-19 booster vaccine uptake was measured using the question: ‘Have you received at least three doses of a COVID-19 vaccine as of today?” These questions were to be answered as either “Yes—have had at least one dose of vaccine/Yes—have had at least three doses of vaccine” or “No—have not had a first dose of vaccine/No—have not had at least three doses of vaccine.” These closed-ended questions indicated vaccine uptake and booster vaccine uptake (27).

The mediating variable was willingness to receive a COVID-19 vaccine. Respondents who responded “No—have not had a first dose of vaccine” were asked the question: “If a COVID-19 vaccine is available, are you willing to receive it?” Respondents were to answer “Willing to receive vaccine,” “Not willing to receive vaccine,” or “Undecided.” Responses of “Not willing to receive vaccine” and ‘Undecided” were assigned as “unwilling” (28, 29).

The main independent variable was knowledge about COVID-19; this was assessed using a six-item questionnaire developed by Zhong et al. (30) and adopted in other similar studies (31, 32). The questionnaire included three questions about the clinical characteristics of the disease (i.e., primary symptoms, availability and effectiveness of treatment, and severity). Two survey questions addressed transmission (i.e., infection through contact with animals and transmission through respiratory droplets), and one item covered prevention and control (i.e., wearing medical masks for prevention). The possible responses were: “Yes,” “No,” or “Do not know.” The knowledge scores were calculated by assigning one point to each correctly answered question and an aggregate score was calculated (range 0–6), with higher scores indicating more knowledge about COVID-19 (31).

We also included comorbidities and sociodemographic factors in the models. Comorbidities were measured through a respondent’s history of hypertension, cardiovascular diseases, diabetes, stroke, autoimmune disease, kidney failure, cancer, gastritis, obesity, chronic obstructive pulmonary disease, and respiratory failure as diagnosed by a medical doctor (33). Sociodemographic factors included gender, age, education, and monthly household income. Each respondent’s educational level was classified based on the Indonesian education system: no schooling, elementary school, junior secondary school, high school, college, and university (28). Household monthly income was classified into four categories based on standard monthly wages in Malang: <1.8 million IDR, 1.8–3 million IDR, 3–4.8 million IDR, and > 4.8 million IDR (34).

### 2.5. Statistical analyses

To ensure that the sample was representative of people living in Malang at large, we generated descriptive statistics (percentages and 95% confidence intervals [CIs]) for the outcomes using cross-sectional weights. Linear regression was used to examine factors associated with knowledge about COVID-19. Logistic regression was performed to examine the association of knowledge about COVID-19 on vaccine uptake. Generalized structural equation modeling (GSEM) was used to examine whether willingness to receive vaccine mediates the association between knowledge about COVID-19 and vaccination uptake. The maximum likelihood (ML) estimator was used to estimate all models; for the probability model, we reported the odds ratio (OR), 95% confidence intervals (95% CIs), and a two-sided value of *p* of <0.05 (35). We used Delta, Sobel, and Monte Carlo tests to determine whether the reduction in the effect of the independent variable after including the mediator variable in the model was significant and, therefore, whether the mediation effect was statistically significant. STATA 17.1 was used to clean and analyze the data. Listwise deletion was used to remove missing data from the analyses, allowing each model to include a different number of participants.

### 2.6. Ethics and consent

The survey was prefaced with a participant information statement and consent form in simple Bahasa (the local language). A trained interviewer read the statement and consent for every participant via the KoboToolbox survey app and confirmed that participants had understood the participant information statement to proceed to the survey; completion of the survey constituted consent. Ethics approval was granted by the Brawijaya University Ethical Board (Reference: 11/EC/KEPK/04/2021).

## 3. Results

### 3.1. Respondent characteristics

Table 1 describes the characteristics of the respondents. The percentage of respondents who reported having received at least one dose of a COVID-19 vaccine was 94.8%, while the percentage of those who reported having received at least three doses was 88.5%. These numbers were higher than the national average for COVID-19 vaccine and booster vaccine uptake. The average age of respondents was 43.6 years old [standard deviation (SD) = 15.0], which is slightly older than the average age of the same age range in the district in 2022. In 2022, the proportion of females in Malang’s population was 49.6%, which is slightly higher than the proportion of female respondents in our study (47.9%). The educational status of respondents was similar to the educational status of Malang’s population in 2022: the largest percentage of the population graduated from high school (35.5%). The greatest number of respondents reported a monthly household income of under 1.8 million rupiahs (49.9%). This percentage was also similar to the 49.6% found in the general population of the district in 2022. The proportion of respondents who reported being unemployed was 8.2%, while in 2022 the open unemployment rate in the district was 7.7% (25). Most respondents reported having no comorbidities (91.8%). Among 10,007 respondents, 4.8% reported having hypertension, 0.9% reported having cardiovascular diseases, 1.1% reported having diabetes, 0.5% reported having chronic obstructive pulmonary disease and respiratory failure, and less than 0.5% reported having had a stroke or autoimmune disease, kidney failure, gastritis, and obesity.

**Table 1:** Respondents’ characteristics.

Variables	% or mean	SD	Min	Max
Received a COVID-19 vaccine	94.8%		0	1
Received a COVID-19 booster vaccine	88.5%		0	1
Willing to receive a COVID-19 vaccine	83.1%		0	1
COVID-19 knowledge score	4.6	1.1	0	6
Age	43.6	15.0	15	99
Sex				
Male	52.1%		0	1
Female	47.9%		0	1
Educational level				
No schooling	1.6%		0	1
Elementary school	31.9%		0	1
Junior secondary school	24.3%		0	1
High school	34.6%		0	1
College	3.3%		0	1
University	4.2%		0	1
Household monthly income (IDR)				
<1.8 million	49.9%		0	1
1.8–3 million	36.1%		0	1
3–4.8 million	11.0%		0	1
>4.8 million	3.0%		0	1
Employment status				
Employed	91.8%		0	1
Unemployed	8.2%		0	1
Comorbidity status				
No comorbidities	91.2%		0	1
Hypertension	4.8%		0	1
Cardiovascular diseases	0.9%		0	1
Diabetes	1.1%		0	1
Stroke	0.4%		0	1
Autoimmune disease	0.1%		0	1
Kidney failure	0.1%		0	1
Chronic obstructive pulmonary disease and respiratory failure	0.5%		0	1
Obesity	0.2%		0	1
Cancer	0.2%		0	1
Gastritis	0.4%		0	1

### 3.2. Respondents’ knowledge regarding COVID-19

Most respondents answered about four of six knowledge items correctly (*M* = 4.60, SD = 1.1). Respondents appeared to be knowledgeable about transmission through respiratory droplets from infected people (92.9% answered correctly, 1.3% incorrectly, and 5.8% reported that they did not know). The highest prevalence of misunderstanding was discovered in the knowledge item regarding infection through eating or having contact with wild animals (Table 2). Only 24.5% correctly answered that transmission does not occur in this way and that the statement was therefore false, while 59.0% believed that it was true and 16.4% responded that they did not know. Most of the respondents (98.2%) correctly replied that wearing a general medical mask aids in prevention, 0.8% answered incorrectly, and 1.0% did not know.

**Table 2:** Respondents’ knowledge about COVID-19.

#	COVID-19 knowledge items	Yes		No		Do not know	
		*N*	%	*N*	%	*N*	%
1	The main clinical symptoms of COVID-19 are fever, fatigue, dry cough, and myalgia.	9,292	92.9%	134	1.3%	581	5.8%
2	There currently is no effective cure for COVID-19, but early symptomatic and supportive treatment can help most patients recover from infection.	8,319	83.1%	278	2.8%	1,410	14.1%
3	Not all persons with COVID-2019 will develop severe cases. Only those who are older adult and have chronic illnesses are more likely to develop severe cases.	7,640	76.3%	1,275	12.7%	1,092	10.9%
4	Eating or having contact with wild animals could result in infection with the COVID-19 virus.	5,908	59.0%	2,455	24.5%	1,644	16.4%
5	The COVID-19 virus spreads via respiratory droplets from infected individuals.	8,433	84.3%	1,146	11.5%	428	4.3%
6	Ordinary citizens can wear general medical masks to prevent infection by the COVID-19 virus.	9,826	98.2%	83	0.8%	98	1.0%

### 3.3. Sociodemographic determinants of COVID-19 knowledge

Knowledge scores varied according to age, educational level, income, and employment status (Table 3). Older respondents were less likely to have accurate information about COVID-19 [*β* = −0.003, 95% CI = −0.005–(−0.002)]. Respondents who were educated at the elementary, junior secondary, high school, college, and university levels were more likely to have accurate information about COVID-19. Respondents with higher economic status were more likely to have accurate information about COVID-19. Unemployed respondents were less likely to have accurate information about COVID-19 [*β* = −0.236, 95% CI = −0.313–(−0.160)]. Gender was not related to knowledge about COVID-19.

**Table 3:** Sociodemographic determinants of COVID-19 knowledge.

Variables	Coef.	SE	Value of *p*	95% CI	
				Lower	Upper
Age	−0.003	0.001	0.000	−0.005	−0.002
Female	−0.004	0.021	0.833	−0.046	0.037
Educational status (reference = no schooling)					
Elementary school	0.332	0.086	0.000	0.163	0.501
Junior secondary school	0.361	0.089	0.000	0.187	0.535
High school	0.428	0.089	0.000	0.253	0.602
College	0.633	0.105	0.000	0.427	0.839
University	0.515	0.101	0.000	0.316	0.714
Household monthly income (IDR) (reference = <1.8 million)					
1.8–3 million	0.172	0.024	0.000	0.125	0.218
3–4.8 million	0.112	0.036	0.002	0.041	0.183
>4.8 million	0.183	0.064	0.004	0.058	0.308
Unemployed	−0.236	0.039	0.000	−0.313	−0.160
Constant	4.298	0.100	0.000	4.102	4.494
Adjusted R^2^	0.025				

### 3.4. Determinants of COVID-19 vaccine uptake and booster vaccine uptake

Table 4 describes the determinants of COVID-19 vaccine uptake. Respondents who had more accurate knowledge about COVID-19 were more likely to be vaccinated (OR = 1.528, 95% CI = 1.428–1.634). Being older (OR = 0.984, 95% CI = 0.977–0.991) and being female (OR = 0.819, 95% CI = 0.676–0.994) were associated with lower COVID-19 vaccination uptake. Respondents with a university education were more likely to be vaccinated than those with no schooling (OR = 2.408, 95% CI = 0.877–6.609). Null associations were found for respondents with elementary school, junior secondary, high school, and college education. Respondents with a monthly household income of more than 4.8 million rupiahs, 3–4.8 million rupiahs, and 1.8–3 million rupiahs were more likely to be vaccinated than respondents with incomes of less than 1.8 million rupiahs. The null association was found for respondents with a monthly household income of 3–4.8 million rupiah. Unemployed respondents were less likely to be vaccinated than employed respondents (OR = 0.348, 95% CI = 0.270–0.448). As expected, most respondents who have comorbidities were less likely to get vaccinated compared to those who did not have comorbidities.

**Table 4:** Logistic regression results of COVID-19 vaccine uptake.

Variables	OR	SE	Value of *p*	95% CI	
				Lower	UPPER
COVID-19 knowledge score	1.528	0.052	0.000	1.428	1.634
Age	0.984	0.004	0.000	0.977	0.991
Female (reference = male)	0.819	0.081	0.043	0.676	0.994
Education status (reference = no schooling)					
Elementary school	0.768	0.201	0.313	0.460	1.283
Junior secondary school	0.985	0.281	0.959	0.564	1.722
High school	1.697	0.499	0.072	0.954	3.021
College	1.520	0.743	0.391	0.583	3.961
University	2.408	1.240	0.088	0.877	6.609
Household monthly income (IDR) (reference = <1.8 million)					
1.8–3 million	1.490	0.175	0.001	1.184	1.876
3–4.8 million	1.331	0.257	0.138	0.912	1.942
>4.8 million	5.296	3.444	0.010	1.480	18.948
Unemployed	0.348	0.045	0.000	0.270	0.448
Comorbidity status (reference = no comorbidities)					
Hypertension	0.498	0.082	0.000	0.360	0.688
Cardiovascular diseases	0.177	0.049	0.000	0.103	0.304
Diabetes	0.140	0.034	0.000	0.087	0.225
Stroke	0.075	0.027	0.000	0.037	0.150
Autoimmune	0.240	0.213	0.108	0.042	1.367
Kidney failure	0.058	0.040	0.000	0.015	0.226
Chronic obstructive pulmonary disease	0.169	0.061	0.000	0.083	0.341
Obesity	1.457	2.097	0.794	0.087	24.486
Cancer	0.111	0.055	0.000	0.042	0.294
Gastritis	0.168	0.076	0.000	0.069	0.407
Constant	8.118	2.978	0.000	3.955	16.661

Table 5 shows the determinants of COVID-19 booster vaccine uptake. Respondents who had more accurate knowledge about COVID-19 were more likely to have received booster vaccination (OR = 1.260, 95% CI = 1.196–1.328). Being older (OR = 0.987, 95% CI = 0.982–0.992) and being female (OR = 0.814, 95% CI = 0.715–0.928) were associated with lower COVID-19 booster vaccination uptake. Respondents with high school and university education were more likely to have received booster vaccination than those with no schooling (OR = 1.535, 95% CI = 0.984–2.397; OR = 2.408, 95% CI = 0.877–6.609, respectively). Null associations were found for respondents with elementary school, junior secondary school, and college education. Respondents with a monthly household income of 3–4.8 million rupiahs and 1.8–3 million rupiahs were more likely to have received booster vaccination than respondents with an income of less than 1.8 million rupiahs. The null association was found for respondents with a monthly household income of more than 4.8 million rupiahs. Unemployed respondents were less likely to have received booster vaccination than employed respondents (OR = 0.522, 95% CI = 0.427–0.638). As expected, respondents who had comorbidities were less likely to have received booster vaccination than those who had no comorbidities.

**Table 5:** Logistic regression results of COVID-19 booster vaccination uptake.

Variables	OR	SE	Value of *p*	95% CI	
				Lower	Upper
COVID-19 knowledge score	1.260	0.034	0.000	1.196	1.328
Age	0.987	0.002	0.000	0.982	0.992
Female	0.814	0.054	0.002	0.715	0.928
Education status (reference = no schooling)					
Elementary school	0.715	0.151	0.111	0.473	1.081
Junior secondary school	1.028	0.230	0.902	0.663	1.593
High school	1.535	0.349	0.059	0.984	2.397
College	1.569	0.513	0.168	0.827	2.978
University	1.764	0.548	0.068	0.960	3.244
Household monthly income (IDR) (reference = <1.8 million)					
1.8–3 million	1.574	0.122	0.000	1.352	1.832
3–4.8 million	1.511	0.195	0.001	1.173	1.947
>4.8 million	1.202	0.262	0.399	0.784	1.842
Unemployed	0.522	0.053	0.000	0.427	0.638
Comorbidity status (reference = no comorbidities)					
Hypertension	0.675	0.086	0.002	0.526	0.868
Cardiovascular diseases	0.437	0.111	0.001	0.266	0.718
Diabetes	0.233	0.050	0.000	0.154	0.354
Stroke	0.165	0.055	0.000	0.087	0.316
Autoimmune	0.710	0.625	0.697	0.127	3.987
Kidney failure	0.194	0.135	0.019	0.049	0.760
Chronic obstructive pulmonary disease	0.347	0.110	0.001	0.187	0.645
Obesity	0.380	0.208	0.077	0.130	1.111
Cancer	0.354	0.168	0.029	0.139	0.899
Gastritis	0.180	0.063	0.000	0.091	0.359
Constant	5.388	1.532	0.000	3.086	9.407

### 3.5. Mediation analyses

**Figure 2:**
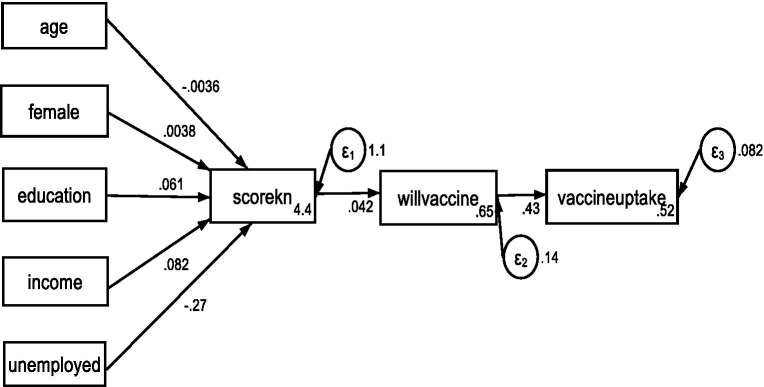
Results of GSEM of the association between COVID-19 knowledge and vaccine uptake with willingness to receive vaccine as the mediation variable.

The indirect effects of knowledge (*scorekn*) on vaccine uptake (*vaccineupatke*) mediated by willingness to receive vaccination (*willvaccine*) were significant (Figure 2; Table 6). The Sobel, Delta, and Monte Carlo tests measuring the statistical significance of indirect effects also showed significance (indirect effect = 0.018, SE = 0.002, *value of p* = 0.000, z-value = 7.123, 95% CI = 0.013–0.023).

**Table 6:** Results of GSEM of the association between COVID-19 knowledge and vaccine uptake with willingness to receive vaccine as the mediation variable.

Variables	Coef.	SE	Value of *p*	95% CI	
				Lower	Upper
COVID-19 knowledge score					
Unemployed	−0.269	0.039	0.000	−0.344	−0.193
Monthly household income	0.082	0.014	0.000	0.054	0.110
Educational level	0.061	0.011	0.000	0.039	0.083
Female	0.004	0.021	0.856	−0.037	0.045
Age	−0.004	0.001	0.000	−0.005	−0.002
Constant	4.441	0.060	0.000	4.323	4.558
Willingness to receive vaccine					
COVID-19 knowledge score	0.042	0.006	0.000	0.030	0.053
Constant	0.646	0.026	0.000	0.594	0.697
Vaccine uptake					
Willingness to receive vaccine	0.426	0.014	0.000	0.399	0.454
Constant	0.523	0.013	0.000	0.499	0.548
Var (e.COVID-19 knowledge score)	1.090	0.015		1.060	1.121
Var (e.Willingness to receive vaccine)	0.138	0.003		0.131	0.145
Var (e.Vaccine uptake)	0.082	0.002		0.078	0.086
Indirect effect	0.018				
Delta test		0.002	0.000	0.013	0.023
Sobel test		0.002	0.000	0.013	0.023
Monte Carlo test		0.002	0.000	0.013	0.023

## 4. Discussion

This study aimed to assess adult Indonesians’ knowledge regarding COVID-19 and COVID-19 booster vaccinations. It found that 94.8% of Malang’s adult population had received at least one dose of COVID-19 vaccine and 88.5% had received at least three doses. These proportions are higher than the national COVID-19 vaccine and booster vaccine uptake. According to the Indonesian Ministry of Health database, the proportion of the population having received at least one dose and at least three doses of COVID-19 vaccine on 18 March 2023 were 86 and 57%, respectively (24). The proportion of individuals in Malang district having received a COVID-19 vaccine booster was also higher than that reported in other countries, including the US (44%), Malaysia (49%), China (57%) (36), and Saudi Arabia (22%) (37).

Our study shows that the level of respondents’ knowledge regarding COVID-19 is relatively high in Malang. Malang residents’ scores were higher than those in prior studies using similar instruments in South Korea and China (31). The regression results show that knowledge scores varied according to age, educational level, income, and employment status, indicating knowledge gaps based on age and socioeconomic status. These results support prior studies showing that members of vulnerable and less affluent groups such as older people, less educated people, people with lower incomes, and unemployed people have less access to information related to COVID-19 (38). Moreover, gender was not related to respondents’ level of knowledge about COVID-19. This finding contrasts with prior studies, many of which show gender gaps in healthcare access with females being disadvantaged compared to males (39). The null findings in this study may reflect similarity in COVID-19 healthcare information-seeking behavior and access to health information as most villagers had the same access to information sources.

Our main results show that higher COVID-19 knowledge scores were associated with higher odds of having received both initial doses of COVID-19 vaccine (OR = 1.528, 95% CI = 1.428–1.634) and booster vaccinations (OR = 1.260, 95% CI = 1.196–1.328). These results confirmed the hypothesis that individuals who have more accurate knowledge regarding COVID-19 are more willing to be vaccinated and are therefore more likely to receive vaccination. The findings support health literacy literature highlighting the key role of individuals’ health knowledge and the importance of information as the foundation of the intention to perform health-related behaviors (8). More specifically, people who have more accurate knowledge about COVID-19 can better understand its health risks, signs, and symptoms as well as the benefits of preventive actions; they also tend to have healthier lifestyles (8). People with less knowledge are more likely to have knowledge deficits about the disease and are therefore less likely to receive vaccination (31).

Our analyses of the mediating variable also show that willingness to receive vaccination mediates the relationship of knowledge to vaccine uptake. These results support other findings that report the benefits of knowledge regarding COVID-19 vaccination as related to willingness to receive vaccination (3). These studies also highlight that people who have sufficient knowledge about a particular vaccine can better understand its potential benefits and importance, which would further shape positive beliefs about the vaccine and strengthen trust in vaccination (7). As such, people with sufficient knowledge do not perceive vaccination as a risky behavior (40). In contrast, those with a lower level of knowledge are more likely to connect vaccines with adverse events and to internalize misinformation about the safety of vaccines, which might increase perceived risk of vaccine side effects (8). Moreover, as one facet of individuals’ health literacy, knowledge about specific health issues can be viewed as a prerequisite for healthy decision-making, including vaccine uptake (41).

This study further found female gender and older age to be related to lower odds of COVID-19 vaccination and booster vaccination uptake. The finding of the effect of gender on COVID-19 vaccine uptake supports prior studies showing that males are less likely to report COVID-19 vaccine hesitancy and more likely to receive COVID-19 vaccination than females (42, 43). Females in China and US have been reported to have limited knowledge regarding the link between COVID-19 vaccination and issues such as pregnancy, fertility, and breastfeeding (44, 45). In addition, males with COVID-19 infections are more likely to be admitted to intensive care unit admission and have higher COVID-19 mortality than females (46). In the present study, higher levels of educational attainment, higher income, and being employed were associated with higher odds of receiving COVID-19 booster vaccination. These findings support prior studies showing the positive association between higher socioeconomic status and the probability of COVID-19 vaccination uptake (47). Among the plausible explanations for this association are that individuals with higher educational levels and incomes may have more trust in biomedical research and government and that they may be more likely to be able to get the logistics regarding vaccine uptake.

The presence of comorbidities, including hypertension, cardiovascular diseases, diabetes, stroke, kidney failure, chronic obstructive pulmonary disease, cancer, and gastritis, was associated with lower vaccination uptake. Prior studies in high-income countries, including the UK and the US (48), have shown higher proportions of COVID-19 vaccine uptake among adults with comorbidities than among healthy individuals. Higher COVID-19 vaccine coverage among people with comorbidities in the UK might be explained by the fact that the UK used risk-based scheduling that prioritized people with comorbidities, e.g., hypertension and type 2 diabetes, for early vaccination (49). A study in China found that only 25.1% of people with diabetes mellitus received COVID-19 vaccination and this proportion was far below the rate in the general population (88%) (50). Furthermore, hospitalized patients with diabetes mellitus and chronic complications had a lower COVID-19 vaccine coverage (11.2%) than those without chronic complications (43.2%). Lack of awareness of the link between chronic complications of diabetes mellitus and the risk and severity of COVID-19 is among the reasons for that low coverage. Another reason is that patients with chronic diabetes mellitus complications were more concerned about the efficacy and safety of COVID-19 vaccination.

People with comorbidities are at greater risk of developing severe COVID-19. Studies have highlighted the importance of COVID-19 boosters among people with comorbidities as comorbidities are among the risk factors for hyporesponsiveness and nondurable response to COVID-19 vaccination (51, 52). The lower COVID-19 booster uptake found in this study among people with comorbidities may be due to the limited availability of data on vaccine safety and efficacy, but noting the increased mortality risk among patients with comorbidities leads to conflicting attitudes toward COVID-19 vaccines. A study using an internet-based survey reported that 1 in 5 respondents with comorbidities were hesitant to receive COVID-19 vaccination (53). Approximately 42% of adults reporting vaccine hesitancy in Central Java, Indonesia stated that having a comorbidity was the reason for their COVID-19 vaccine hesitancy (54). The Indonesian Ministry of Health regulates the provision of COVID-19 vaccine for older people and people with comorbidities and has listed the conditions in which the vaccine cannot be administered to patients (55). This regulation is not accompanied by a good information source for the general population. This is cause for great concern given that individuals with cancer and other serious comorbidities have an increased risk of mortality if they contact COVID-19. Information on vaccine efficacy and safety is related to higher acceptance. Providing health-related social media forums that rapidly disseminate accurate information about COVID-19 vaccination, especially for high-risk populations, may play an important role in increasing vaccine uptake.

### 4.1. Limitations

Several limitations of this study should be acknowledged. First, the analysis used average knowledge scores, so the specific effects of accurate responses to each individual item were not examined. Second, this study did not extensively explore other attitudinal factors associated with COVID-19 behaviors, such as perceived barriers or other communication factors including information seeking, media usage, and information processing, that may have influenced public knowledge. Third, some of the variables in this study were based on retrospective data, especially regarding respondents’ vaccination uptake and respondents’ histories of comorbidities. These data were thus subject to recall bias. Researchers conducting further studies may wish to use medical record data collected from primary healthcare centers or hospitals to address the issue of recall bias.

### 4.2. Implications

This study has important implications for policymakers and health practitioners with valuable insights into how to create an effective strategy to increase COVID-19 vaccination uptake in the district and LMICs with similar health contexts. First, improving knowledge of the vaccine itself, including its efficacy and safety, is not enough to improve COVID-19 vaccine and booster vaccine uptake. Policymakers and health practitioners need to improve public knowledge of COVID-19 in general by acknowledging and discussing their concerns about the disease. Although most of the extant literature has focused on knowledge specifically related to COVID-19 vaccines to improve coverage (55), some studies have highlighted the importance of knowledge about and positive attitudes toward the disease itself in increasing vaccine acceptance (56–58). To tackle vaccine hesitancy and increase uptake, policymakers in Indonesia and other LMICs thus need to design strategies to deliver accurate information not only regarding COVID-19 vaccines but also regarding the disease in general. Furthermore, rumors and misconceptions about COVID-19 and COVID-19 vaccines, especially on social media, should be dismissed and people should be exposed to scientific facts to improve COVID-19 vaccine uptake.

Second, our study shows heterogeneity in COVID-19 vaccine uptake across demographics and socioeconomic characteristics; older people, those with lower levels of educational attainment, those with lower incomes, and those who were unemployed had lower vaccine uptake than others. However, knowledge of COVID-19 and COVID-19 vaccines is lower among individuals with lower socioeconomic status, and providing information to these individuals is more challenging due to limited public health and healthcare services and other infrastructural issues such as the digital divide (58–61). Despite improving accessibility of vaccination programs to those specific socioeconomic groups, introducing more public health strategies to deliver accurate information is thus important to address communication inequalities and design public health communications that will more effectively reduce the existing disparities across segments of the population.

Finally, our findings have important implications for the rollout of booster vaccines. Despite robust immune responses after two doses of COVID-19 vaccine, comorbidities are strongly associated with hyporesponsiveness to COVID-19 vaccination. Booster vaccination is thus required to maintain high levels of protective antibodies in individuals with comorbidities. Our findings showing lower booster uptake among people with comorbidities suggest that interventions to improve access and health literacy need to be provided for these individuals in particular.

### 4.3. Conclusion

In conclusion, this study found significant positive associations between COVID-19 knowledge and vaccine uptake. Our findings suggest that interventions and public health programs aiming to improve knowledge, attitudes, and perceptions regarding COVID-19 vaccination can be implemented to improve vaccine uptake. Furthermore, our findings may contribute to developing a strategy for controlling the COVID-19 pandemic by addressing other determinants of vaccination uptake, including age, gender, and socioeconomic status.

## Data availability statement

The original contributions presented in the study are included in the article/supplementary material, further inquiries can be directed to the corresponding author.

## Ethics statement

The studies involving human participants were reviewed and approved by the Brawijaya University Ethical Board (Reference: 11/EC/KEPK/04/2021). The participants provided their written informed consent to participate in this study. Written informed consent to participate in this study was provided by the participants' legal guardian/next of kin.

## Author contributions

SS and AM prepared the study design, collected data, conducted data analyses, and reviewed the manuscript. All authors contributed to the article and approved the submitted version.

## Funding

This study was funded by the Endowment Fund for Education (LPDP), Ministry of Finance, Indonesia, based on Decision Letter Number KEP-43/LPDP/2019 dated June 26, 2019; Decision Letter Number KEP-20/LPDP/2021 dated March 10, 2021; Cooperative Agreement to participate in the International Collaboration-Productive Open Call Research Funding scheme, based on Number PRJ-71/LPDP/2021 dated April 14, 2021, and Number: 01/DIPI/2021 dated April 14, 2021, for Research Grant Number RISPRO/KI/B1/TKL/5/15129/1/2020.

## Conflict of interest

The authors declare that the research was conducted in the absence of any commercial or financial relationships that could be construed as a potential conflict of interest.

## Publisher’s note

All claims expressed in this article are solely those of the authors and do not necessarily represent those of their affiliated organizations, or those of the publisher, the editors and the reviewers. Any product that may be evaluated in this article, or claim that may be made by its manufacturer, is not guaranteed or endorsed by the publisher.

## References

[1] Machingaidze S Wiysonge CS. Understanding COVID-19 vaccine hesitancy. Nat Med. (2021) 27:1338–9. doi: 10.1038/s41591-021-01459-7 34272500

[2] Smith LE Amlôt R Weinman J Yiend J Rubin GJ. A systematic review of factors affecting vaccine uptake in young children. Vaccine. (2017) 35:6059–69. doi: 10.1016/j.vaccine.2017.09.046, PMID: 28974409

[3] Neely SR Eldredge C Ersing R Remington C. Vaccine hesitancy and exposure to misinformation: a survey analysis. J Gen Intern Med. (2022) 37:179–87. doi: 10.1007/s11606-021-07171-z 34671900 PMC8528483

[4] Ahiakpa JK Cosmas NT Anyiam FE Enalume KO Lawan I Gabriel IB. COVID-19 vaccines uptake: public knowledge, awareness, perception and acceptance among adult Africans. PLoS One. (2022) 17:e0268230. doi: 10.1371/journal.pone.0268230, PMID: 35648745 PMC9159554

[5] Hudson A Montelpare WJ. Predictors of vaccine hesitancy: implications for COVID-19 public health messaging. Int J Environ Res Public Health. (2021) 18:8054. doi: 10.3390/ijerph18158054, PMID: 34360345 PMC8345367

[6] Okan O Messer M Levin-Zamir D Paakkari L Sørensen K. Health literacy as a social vaccine in the COVID-19 pandemic. Health Promot Int. (2022) 1–9. doi: 10.1093/heapro/daab197, PMID: 35022721 PMC8807235

[7] Zheng H Jiang S Wu Q. Factors influencing COVID-19 vaccination intention: the roles of vaccine knowledge, vaccine risk perception, and doctor-patient communication. Patient Educ Couns. (2022) 105:277–83. doi: 10.1016/j.pec.2021.09.023, PMID: 34565643 PMC8450210

[8] Miller LMS Gee PM Katz RA. The importance of understanding COVID-19: the role of knowledge in promoting adherence to protective behaviors. Front Public Health. (2021) 9:581497. doi: 10.3389/fpubh.2021.581497, PMID: 33889557 PMC8055953

[9] Klahr D Kotovsky K. Complex information processing: The impact of Herbert a. Simon. London: Psychology Press (2013).

[10] Suen LK So ZYY Yeung SKW Lo KYK Lam SC. Epidemiological investigation on hand hygiene knowledge and behaviour: a cross-sectional study on gender disparity. BMC Public Health. (2019) 19:401–14. doi: 10.1186/s12889-019-6705-5, PMID: 30975130 PMC6460727

[11] Bults M Beaujean DJMA Richardus JH Voeten HACM. Perceptions and behavioral responses of the general public during the 2009 influenza a (H1N1) pandemic: a systematic review. Disaster Med Public Health Prep. (2015) 9:207–19. doi: 10.1017/dmp.2014.160, PMID: 25882127

[12] Zickfeld JH Schubert TW Herting AK Grahe J Faasse K. Correlates of health-protective behavior during the initial days of the COVID-19 outbreak in Norway. Front Psychol. (2020) 11:564083. doi: 10.3389/fpsyg.2020.564083, PMID: 33123045 PMC7573186

[13] Moreland A Herlihy C Tynan MA Sunshine G McCord RF Hilton C. Timing of state and territorial COVID-19 stay-at-home orders and changes in population movement—United States, March 1–May 31, 2020. Morb Mortal Wkly Rep. (2020) 69:1198–203. doi: 10.15585/mmwr.mm6935a2, PMID: 32881851 PMC7470456

[14] Clements JM. Knowledge and behaviors toward COVID-19 among US residents during the early days of the pandemic: cross-sectional online questionnaire. JMIR Public Health Surveill. (2020) 6:e19161. doi: 10.2196/19161, PMID: 32369759 PMC7212816

[15] Mutombo PN Fallah MP Munodawafa D Kabel A Houeto D Goronga T. COVID-19 vaccine hesitancy in Africa: a call to action. Lancet Glob Health. (2022) 10:e320–1. doi: 10.1016/S2214-109X(21)00563-5, PMID: 34942117 PMC8687664

[16] Simas C Larson HJ. Overcoming vaccine hesitancy in low-income and middle-income regions. Nat Rev Dis Primers. (2021) 7:41. doi: 10.1038/s41572-021-00279-w, PMID: 34112811

[17] Richardson S Hirsch JS Narasimhan M Crawford JM McGinn T Davidson KW. Presenting characteristics, comorbidities, and outcomes among 5700 patients hospitalized with COVID-19 in the New York City area. JAMA. (2020) 323:2052–9. doi: 10.1001/jama.2020.6775 32320003 PMC7177629

[18] Myers LC Parodi SM Escobar GJ Liu VX. Characteristics of hospitalized adults with COVID-19 in an integrated health care system in California. JAMA. (2020) 323:2195–8. doi: 10.1001/jama.2020.7202, PMID: 32329797 PMC7182961

[19] Mohseni H Kiran A Khorshidi R Rahimi K. Influenza vaccination and risk of hospitalization in patients with heart failure: a self-controlled case series study. Eur Heart J. (2017) 38:326–33. doi: 10.1093/eurheartj/ehw411, PMID: 27660378 PMC5837634

[20] Gallone MS Infantino V Ferorelli D Stefanizzi P de Nitto S Tafuri S. Vaccination coverage in patients affected by chronic diseases: a 2014 cross-sectional study among subjects hospitalized at Bari Policlinico General Hospital. Am J Infect Control. (2018) 46:e9–e11. doi: 10.1016/j.ajic.2017.10.004 29167031

[21] Jiang N Gu P Sun X Han H Liu W Song N. Acceptance of COVID-19 vaccines in patients with chronic diseases: a cross-sectional study. J Clin Nurs. (2022) 31:3286–300. doi: 10.1111/jocn.16284, PMID: 35285111 PMC9115274

[22] Zhao Y du J Li Z Xu Z Wu Y Duan W. It is time to improve the acceptance of COVID-19 vaccines among people with chronic diseases: a systematic review and meta-analysis. J Med Virol. (2023) 95:e28509. doi: 10.1002/jmv.28509, PMID: 36655758

[23] Indonesian Ministry of Health. National COVID-19 Vaccine Coverage. Jakarta: Indonesian Ministry of Health (2023).

[24] Malang District Health Office. Malang COVID-19 vaccine coverage. Malang: Malang District Health Office (2023).

[25] Malang District Government. Malang statistic year report 2022. Malang: Malang District Government (2023).

[26] Lakshminarasimhappa M. Web-based and smart mobile app for data collection: kobo toolbox/kobo collect. J. Indian Lib. Assoc. (2022) 57:72–9.

[27] Rikitu Terefa D Shama AT Feyisa BR Desisa AE Geta ET Cheme MC. COVID-19 vaccine uptake and associated factors among health professionals in Ethiopia. Infect. Drug Resist. (2021) 14:5531–41. doi: 10.2147/IDR.S344647, PMID: 34984008 PMC8702775

[28] Song S Zang S Gong L Xu C Lin L Francis MR. Willingness and uptake of the COVID-19 testing and vaccination in urban China during the low-risk period: a cross-sectional study. BMC Public Health. (2022) 22:1–13. doi: 10.1186/s12889-022-12969-5 35313843 PMC8935604

[29] Nguyen LH Joshi AD Drew DA Merino J Ma W Lo CH. Self-reported COVID-19 vaccine hesitancy and uptake among participants from different racial and ethnic groups in the United States and United Kingdom. Nat Commun. (2022) 13:636. doi: 10.1038/s41467-022-28200-3, PMID: 35105869 PMC8807721

[30] Zhong B-L Luo W Li HM Zhang QQ Liu XG Li WT. Knowledge, attitudes, and practices towards COVID-19 among Chinese residents during the rapid rise period of the COVID-19 outbreak: a quick online cross-sectional survey. Int J Biol Sci. (2020) 16:1745–52. doi: 10.7150/ijbs.45221, PMID: 32226294 PMC7098034

[31] Lee M Kang B-A You M. Knowledge, attitudes, and practices (KAP) toward COVID-19: a cross-sectional study in South Korea. BMC Public Health. (2021) 21:1–10. doi: 10.1186/s12889-021-10285-y 33546644 PMC7863060

[32] Qutob N Awartani F. Knowledge, attitudes and practices (KAP) towards COVID-19 among Palestinians during the COVID-19 outbreak: a cross-sectional survey. PLoS One. (2021) 16:e0244925. doi: 10.1371/journal.pone.0244925, PMID: 33400722 PMC7785223

[33] Adab P Haroon S O’Hara ME Jordan RE. Comorbidities and COVID-19. BMJ. (2022). doi: 10.1136/bmj.o1431 35705219

[34] Sujarwoto S Holipah H Maharani A. A cross-sectional study of knowledge, attitudes, and practices concerning COVID-19 outbreaks in the general population in Malang District, Indonesia. Int J Environ Res Public Health. (2022) 19:4287. doi: 10.3390/ijerph19074287, PMID: 35409968 PMC8998605

[35] Menard S. Applied logistic regression analysis. Thousand Oaks, CA: Sage (2002).

[36] Shah A Coiado OC. COVID-19 vaccine and booster hesitation around the world: a literature review. Front Med. (2023) 9:3994. doi: 10.3389/fmed.2022.1054557 PMC9878297 36714110

[37] Alshahrani NZ Alsabaani AA Ridda I Rashid H Alzahrani F Almutairi TH. Uptake of COVID-19 booster dose among Saudi Arabian population. Medicina. (2022) 58:972. doi: 10.3390/medicina58070972, PMID: 35888690 PMC9323634

[38] Anjorin AA Odetokun IA Abioye AI Elnadi H Umoren MV Damaris BF. Will Africans take COVID-19 vaccination? PLoS One. (2021) 16:e0260575. doi: 10.1371/journal.pone.0260575, PMID: 34851998 PMC8635331

[39] Pinchoff J Santhya KG White C Rampal S Acharya R Ngo TD. Gender specific differences in COVID-19 knowledge, behavior and health effects among adolescents and young adults in Uttar Pradesh and Bihar, India. PLoS One. (2020) 15:e0244053. doi: 10.1371/journal.pone.0244053, PMID: 33332461 PMC7746145

[40] Yaqub O Castle-Clarke S Sevdalis N Chataway J. Attitudes to vaccination: a critical review. Soc Sci Med. (2014) 112:1–11. doi: 10.1016/j.socscimed.2014.04.018 24788111

[41] Castro-Sánchez E Chang PWS Vila-Candel R Escobedo AA Holmes AH. Health literacy and infectious diseases: why does it matter? Int J Infect Dis. (2016) 43:103–10. doi: 10.1016/j.ijid.2015.12.019, PMID: 26751238

[42] Dror AA Eisenbach N Taiber S Morozov NG Mizrachi M Zigron A. Vaccine hesitancy: the next challenge in the fight against COVID-19. Eur J Epidemiol. (2020) 35:775–9. doi: 10.1007/s10654-020-00671-y, PMID: 32785815 PMC8851308

[43] Khubchandani J Sharma S Price JH Wiblishauser MJ Sharma M Webb FJ. COVID-19 vaccination hesitancy in the United States: a rapid national assessment. J Community Health. (2021) 46:270–7. doi: 10.1007/s10900-020-00958-x 33389421 PMC7778842

[44] Nguyen K.H. Srivastav A. Razzaghi H. Williams W. Lindley M.C. Jorgensen C.. COVID-19 vaccination intent, perceptions, and reasons for not vaccinating among groups prioritized for early vaccination—United States, September and December 2020. (2021), Hoboken, HJ: Wiley Online Library, 21, 1650–1656. 33788992 10.1111/ajt.16560 PMC8250395

[45] Xu B Gao X Zhang X Hu Y Yang H Zhou YH. Real-world acceptance of COVID-19 vaccines among healthcare workers in perinatal medicine in China. Vaccine. (2021) 9:704. doi: 10.3390/vaccines9070704, PMID: 34199143 PMC8310137

[46] Peckham H de Gruijter NM Raine C Radziszewska A Ciurtin C Wedderburn LR. Male sex identified by global COVID-19 meta-analysis as a risk factor for death and ITU admission. Nat Commun. (2020) 11:6317. doi: 10.1038/s41467-020-19741-6, PMID: 33298944 PMC7726563

[47] Malesza M Bozym M. Factors influencing COVID-19 vaccination uptake in an elderly sample in Poland. medRxiv. (2021). doi: 10.1101/2021.03.21.21254047

[48] Nguyen LH Joshi AD Drew DA Merino J Ma W Lo C-H. Racial and ethnic differences in COVID-19 vaccine hesitancy and uptake. medrxiv. (2021). doi: 10.1101/2021.02.25.21252402

[49] UK health security agency, coronavirus (COVID-19) vaccination programme in the UK. London: UK Health Security Agency (2020).

[50] Xu J Chen S Wang Y Duan L Li J Shan Y. Prevalence and determinants of COVID-19 vaccination uptake were different between Chinese diabetic inpatients with and without chronic complications: a cross-sectional survey. Vaccine. (2022) 10:994. doi: 10.3390/vaccines10070994 PMC9317053 35891159

[51] Søgaard OS Reekie J Johansen IS Nielsen H Benfield T Wiese L. Characteristics associated with serological COVID-19 vaccine response and durability in an older population with significant comorbidity: the Danish Nationwide ENFORCE study. Clin Microbiol Infect. (2022) 28:1126–33. doi: 10.1016/j.cmi.2022.03.003, PMID: 35283313 PMC8913025

[52] Mallah N Pardo-Seco J López-Pérez LR González-Pérez JM Rosón B Otero-Barrós MT. Effectiveness of COVID-19 vaccine booster in the general population and in subjects with comorbidities. A population-based study in Spain. Environ Res. (2022) 215:114252. doi: 10.1016/j.envres.2022.114252, PMID: 36096168 PMC9462926

[53] Tsai R Hervey J Hoffman K Wood J Johnson J Deighton D. COVID-19 vaccine hesitancy and acceptance among individuals with cancer, autoimmune diseases, or other serious comorbid conditions: cross-sectional, internet-based survey. JMIR Public Health Surveill. (2022) 8:e29872. doi: 10.2196/29872 34709184 PMC8734610

[54] Utami A Margawati A Pramono D Nugraheni A Pramudo S. Determinant factors of COVID-19 vaccine hesitancy among adult and elderly population in Central Java, Indonesia. Patient Prefer Adherence. (2022) 16:1559–70. doi: 10.2147/PPA.S365663, PMID: 35789823 PMC9250340

[55] Ministry of Health, Republic of Indonesia. Regulation number HK.02.02/I/368/2021 regarding COVID-19 vaccination on older people, people with comorbidity and COVID-19 survivals or Pelaksanaan Vaksinasi COVID-19 pada Kelompok Sasaran Lansia, Komorbid dan Penyintas COVID-19. Jakarta: Ministry of Health, Republic of Indonesia (2021).

[56] Adane M Ademas A Kloos H. Knowledge, attitudes, and perceptions of COVID-19 vaccine and refusal to receive COVID-19 vaccine among healthcare workers in northeastern Ethiopia. BMC Public Health. (2022) 22:128. doi: 10.1186/s12889-021-12362-8, PMID: 35042476 PMC8765812

[57] Mahmud S Mohsin M Khan IA Mian AU Zaman MA. Knowledge, beliefs, attitudes and perceived risk about COVID-19 vaccine and determinants of COVID-19 vaccine acceptance in Bangladesh. PLoS One. (2021) 16:e0257096. doi: 10.1371/journal.pone.0257096, PMID: 34499673 PMC8428569

[58] Trucchi C Costantino C Restivo V Bertoncello C Fortunato F Tafuri S. Immunization campaigns and strategies against human papillomavirus in Italy: the results of a survey to regional and local health units representatives. Biomed Res Int. (2019) 2019:1–8. doi: 10.1155/2019/6764154, PMID: 31355274 PMC6637711

[59] Hammour KA Farha RA Manaseer Q Al-Manaseer B. Factors affecting the public’s knowledge about COVID-19 vaccines and the influence of knowledge on their decision to get vaccinated. J Am Pharm Assoc. (2022) 62:309–16. doi: 10.1016/j.japh.2021.06.021, PMID: 34301497 PMC8259061

[60] Bono SA Faria de Moura Villela E Siau CS Chen WS Pengpid S Hasan MT. Factors affecting COVID-19 vaccine acceptance: an international survey among low-and middle-income countries. Vaccine. (2021) 9:515. doi: 10.3390/vaccines9050515, PMID: 34067682 PMC8157062

[61] Sujarwoto S Maharani A. Sociodemographic characteristics and health access associated with COVID-19 infection and death: a cross-sectional study in Malang District, Indonesia. BMJ Open. (2022) 12:e052042. doi: 10.1136/bmjopen-2021-052042, PMID: 35613769 PMC9130669

